# Tempo and mode of allopatric divergence in the weakly electric fish *Sternopygus dariensis* in the Isthmus of Panama

**DOI:** 10.1038/s41598-019-55336-y

**Published:** 2019-12-11

**Authors:** Celestino Aguilar, Matthew J. Miller, Jose R. Loaiza, Rigoberto González, Rüdiger Krahe, Luis F. De León

**Affiliations:** 1 Centro de Biodiversidad y Descubrimiento de Drogas, Instituto de Investigaciones Científicas y Servicios de Alta Tecnología (INDICASAT AIP), P. O. Box 0843-01103, Panamá, República de Panamá; 2 Department of Biotechnology, Acharya Nagarjuna University, Guntur, India; 3 Smithsonian Tropical Research Institute, Balboa Ancón, P.O. Box 0843-03092, Panamá, República de Panamá; 4 Department of Biology, University of Massachusetts Boston, Boston, MA USA; 5 Sam Noble Oklahoma Museum of Natural History and Department of Biology, University of Oklahoma, Norman, OK USA; 6 Programa Centroamericano de Maestría en Entomología, Universidad de Panamá, Panamá, República de Panamá; 7 Institut für Biologie, Humboldt-Universität zu Berlin, Berlin, Germany; 8 Department of Biology, McGill University, Montreal, QC Canada

**Keywords:** Population genetics, Biodiversity

## Abstract

Spatial isolation is one of the main drivers of allopatric speciation, but the extent to which spatially-segregated populations accumulate genetic differences relevant to speciation is not always clear. We used data from ultraconserved elements (UCEs) and whole mitochondrial genomes (i.e., mitogenomes) to explore genetic variation among allopatric populations of the weakly electric fish *Sternopygus dariensis* across the Isthmus of Panama. We found strong genetic divergence between eastern and western populations of *S. dariensis*. Over 77% of the UCE loci examined were differentially fixed between populations, and these loci appear to be distributed across the species’ genome. Population divergence occurred within the last 1.1 million years, perhaps due to global glaciation oscillations during the Pleistocene. Our results are consistent with a pattern of genetic differentiation under strict geographic isolation, and suggest the presence of incipient allopatric species within *S. dariensis*. Genetic divergence in *S. dariensis* likely occurred *in situ*, long after the closure of the Isthmus of Panama. Our study highlights the contribution of spatial isolation and vicariance to promoting rapid diversification in Neotropical freshwater fishes. The study of spatially-segregated populations within the Isthmus of Panama could reveal how genetic differences accumulate as allopatric speciation proceeds.

## Introduction

The closure of the Isthmus of Panama is one of the main drivers of Neotropical diversification. On the one hand, the rise of the Isthmus resulted in immediate reduction in gene flow between marine organisms on the two sides of the Isthmus^1–4^. On the other, the exposure of the land bridge facilitated dispersal and colonization in both terrestrial^5^ and freshwater organisms^6,7^. In addition, global events such as the Pleistocene glaciations^8^ have facilitated the expansion and contraction of local populations via changes in sea level^9–11^. Together, these events have defined the current spatial structure of the Isthmian populations^6,12–14^, with implications for allopatric divergence across isolated populations.

Accordingly, spatial isolation of populations along and across the Isthmus of Panama is expected to facilitate the accumulation of genetic differences leading to the formation of new species^15–19^. However, the extent to which spatially segregated populations accumulate genetic differences relevant to allopatric divergence is not always clear. In addition, if divergence occurs, its historical context might be difficult to define, given the dynamic nature of the rise of the Isthmus of Panama. For instance, allopatric divergence might proceed randomly across the range of segregated populations, or it might be driven by multiple dispersal and colonization events^6–21^. Furthermore, divergence of seemingly isolated populations might be influenced by selective (i.e., local adaptation) and random processes (i.e., drift) that are difficult to disentangle if relying on low numbers of molecular markers^22^. Here, we explore these issues by quantifying genetic variation at both nuclear loci linked to ultraconserved elements (UCEs^23,24^; and whole mitochondrial genomes across geographically segregated populations of the weakly electric fish, *Sternopygus dariensis*, in the Isthmus of Panama.

*Sternopygus dariensis* (Meek & Hildebrand, 1916) is a geographically unique species within the Blue-green knifefish (*S. aequilabiatus*; Humboldt, 1805) species complex. Similar to other Neotropical electric fishes^25^, this *Sternopygus* complex originated in South America, but it has since colonized the Isthmus of Panama. Although it has not been resolved whether *S. aequilabiatus* and *S. dariensis* are allospecies or distinct species, here we consider *S. dariensis* as an independent species following Hulen *et al*.^26^ and Albert^27^. However, our inferences do not change if we consider *S. dariensis* as synonym of *S. aequilabiatus* as suggested by Maldonado *et al*.^28^. *Sternopygus dariensis* is narrowly distributed from the Pacific slope of Colombia to the Tabasará River in western Panama^26^, which represents the effective range limit of the species. As with other weakly electric fishes, *S. dariensis* has an elongate eel-shaped body and the ability to produce electric organ discharges (EOD) used for electrolocation and communication^29,30^. Interestingly, despite evidence for genetic variation in other species of the genus *Sternopygus*^31,32^ no population-level analysis has been performed within the Isthmus of Panama. Thus, quantifying genetic variation across Isthmian populations of *S. dariensis* will help inform the tempo and mode of allopatric divergence in Neotropical freshwater fishes.

By integrating available data from mitogenomes as well as UCEs, we examine i) the historical factors driving allopatric divergence across spatially segregated populations, and ii) the genetic consequences of allopatric divergence in the weakly electric fish *S. dariensis* across the Isthmus of Panama.

## Results

### Ultraconserved elements (UCEs)

**Figure 1:**
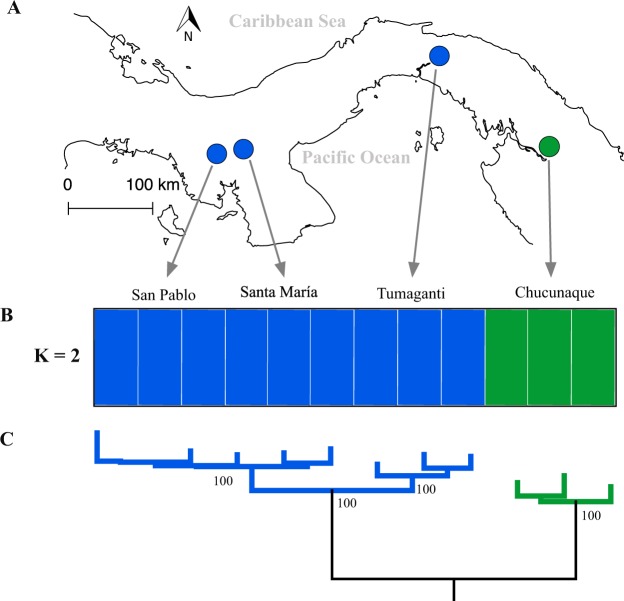
Phylogenetic reconstruction of allopatric populations of *S. dariensis* based on UCE data. The figure shows sampling sites (**A**), population structure with K = 2 representing the highest posterior probabilities as implemented in STRUCTURE and CLUMPPAK, for 118 SNPs (**B**), and maximum likelihood tree inferred by RAxML, for 98 UCEs sequences. Numbers at the nodes are bootstrap values (**C**).

We recovered 150 UCE loci (32 were invariant) that had an average length of 861 bp shared across individuals of *S. dariensis*. The 118 variant loci contained 285 single nucleotide polymorphisms (SNPs), ranging from 1 to 8 SNPs per locus. The complete dataset including the outgroup *Eigenmannia humboldtii* had 98 enriched UCE loci with a total alignment length of 85 028 bp. UCE raw read data are available on NCBI SRA SRP071703 (BioProject PRJNA480353, see Table 1 for BioSample numbers). Overall, our RAxML phylogeny analysis using 98 UCEs showed high bootstrap support (100%) for one western and one eastern clade, comprising samples from San Pablo, Santa María, and Tumaganti, and samples from Chucunaque, respectively (Fig. 1C).

**Table 1:** Sample information for *S. dariensis* and other Gymnotiformes included in the present study.

Sample ID	Species	Region	River	Genbank		Reference
				BioSample	mtDNA	
PB05	*Sternopygus dariensis*	eastern	Chucunaque	SAMN09637294	MK530706	Present study
PB06	*Sternopygus dariensis*	eastern	Chucunaque	SAMN09637295	MH399590	Aguilar *et al*. 2019
PB07	*Sternopygus dariensis*	eastern	Chucunaque	SAMN09637296	—	Present study
Tu218	*Sternopygus dariensis*	western	Tumaganti	SAMN09637297	MK530707	Present study
Tu219	*Sternopygus dariensis*	western	Tumaganti	SAMN09637298	MH605315	Present study
Tu221	*Sternopygus dariensis*	western	Tumaganti	SAMN09637299	—	Present study
RSM01	*Sternopygus dariensis*	western	Santa María	SAMN09637300	MH605309	Present study
RSM02	*Sternopygus dariensis*	western	Santa María	SAMN09637301	MH605310	Present study
RSM03	*Sternopygus dariensis*	western	Santa María	SAMN09637302	MH605311	Present study
RSP02	*Sternopygus dariensis*	western	San Pablo	SAMN09637303	MH605312	Present study
RSP03	*Sternopygus dariensis*	western	San Pablo	SAMN09637304	MH605313	Present study
RSP04	*Sternopygus dariensis*	western	San Pablo	SAMN09637305	MH605314	Present study
Tu227	*Eigenmannia humboldtii*	outgroup	Tumaganti	SAMN09637306	—	Present study
PB01	*Eigenmannia humboldtii*	outgroup	Chucunaque	SAMN09637307	—	Present study
—	*Sternopygus arenatus*	outgroup	—	—	KX058571	Elbassiouny *et al*. 2016
—	*Sternopygus macrurus*	outgroup			MH263671	Rincón-Sandoval *et al*. 2018
—	*Eigenmannia humboldtii*	outgroup			MH263668	Rincón-Sandoval *et al*. 2018
—	*Eigenmannia sp*.	outgroup	—	—	AB054131	Saitoh *et al*. 2003
—	*Apteronotus rostratus*	outgroup			MH399592	Aguilar *et al*. 2019

Genbank short-read archive (BioSample) for ultraconserved elements (UCEs) and accession numbers for mitogenomes (mtDNA) are also provided.

Similar results were obtained with the Bayesian clustering analyses (based on the 118 SNPs), which revealed high posterior probability for two well-defined independent clusters (K = 2), as identified by maximum ΔK value (Fig. 1B). These two clusters corresponded to western (Santa María, San Pablo and Tumaganti), and eastern (Chucunaque) Panama, respectively.

**Figure 2:**
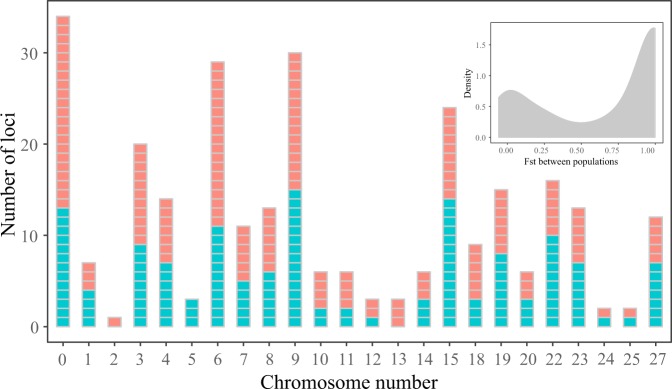
Chromosomal position of SNPs linked to UCEs in *S. dariensis*. Pink bars show highly differentiated SNPs between eastern and western populations within the Isthmus of Panama. Blue bars show non-differentiated SNPs. Chromosome mapping was done using the channel catfish reference genome. Chromosome “0” indicates a set of SNPs that did not map to any of the known chromosomes. The upper right inset shows the frequency distribution of *F*_ST_ values between eastern and western populations across the entire SNP dataset.

When examining genetic divergence between clades, we identified a total of 91 (77%) UCE loci containing 150 fixed SNPs. All UCE loci were successfully mapped to the available genomic scaffolds of the electric eel (*Electrophorus electricus*), and 83 loci (containing 129 fixed SNPs) were mapped to the channel catfish (*Ictalurus punctatus*) genome (Fig. 2; Supplementary Table S1). These 129 fixed SNPs were located in 22 of the 29 channel catfish chromosomes, and ranged from 1 to 18 SNPs per chromosome, with chromosome 6 showing the highest number of fixed SNPs (Fig. 2). In addition, 21 fixed SNPs did not map to any of the known channel catfish chromosomes. Across the complete data matrix (285 SNPs), only seven chromosomes (5, 16, 17, 21, 26, 28 and 29) did not show fixed SNPs (Fig. 2). The distribution of fixed SNPs across the electric eel scaffolds was widespread and appeared to show similar positions with respect to their location on the channel catfish chromosomes (Supplementary Table S1). In addition, the frequency distribution of *F*_ST_ estimates between clades was highly skewed toward large values (Mean = 0.65, Median = 1.0, Skewness = −0.62; Fig. 2). Finally, our analysis of outlier loci using BayeScan 2.1^33^ failed to detect loci under directional selection (Mean *q*-val = 0.90). By contrast, the PCAdapt analysis identified 12 potential outlier loci associated with differences between the two lineages, at FDR of 0.05 (Fig. S1). These SNPs occurred over 11 separate chromosomes of the channel catfish genome.

### Mitogenomes

**Figure 3:**
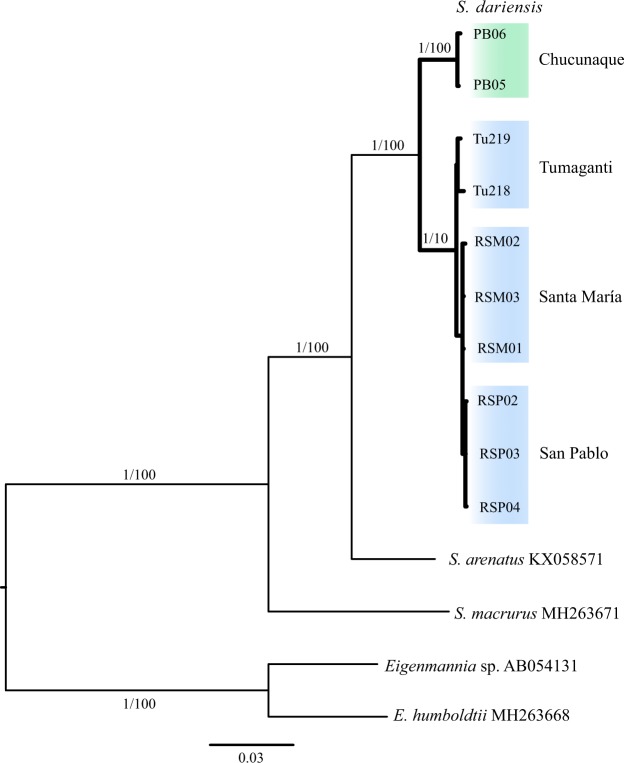
Phylogenetic relationships among *S. dariensis* based on RAxML and MrBayes. The phylogeny represents the best-scoring maximum likelihood tree based on 12 PCGs. Branch lengths are mean estimates. Numbers at the nodes are posterior probabilities and bootstrap values. The scale bar indicates relative branch lengths.

We recovered a total of 9 complete mitogenomes: 8 from the present study (GenBank accession nos. MH605309-MH605315 and MK530706) and one assembled previously (MH399590^34^) (Table 1). We also were able to retrieve a partial mitogenome from 1 individual from Tumaganti (GenBank accession no. MK530707). Both maximum likelihood and Bayesian phylogenetic analyses based on a concatenated dataset of 12 protein-coding genes (PCGs) derived from whole mitogenomes yielded a monophyletic relationship among samples of *S. dariensis* collected in Panama (Fig. 3). In agreement with the UCE results, we detected two highly supported (BS, 100%; PP, 1.0) phylogenetic clades within *S. dariensis* (Fig. 1C). One clade comprised samples from the three western populations (San Pablo, Santa María and Tumaganti rivers), and the other encompassed samples from the eastern population of the Chucunaque River (Fig. 3). Furthermore, the most western populations of *S. dariensis*, San Pablo and Santa María and Tumaganti, showed similar genetic distances among them (~0.18%), but the greatest genetic distance (2.83%, SE = 0.25%) from the eastern population of the Chucunaque River (Table 2). Across species, we found high levels of genetic divergence between *S. dariensis* and its most closely related species, *S. arenatus* (6.6%, SE = 0.91%) (Table 3).

**Table 2:** Pairwise uncorrected p-distances between populations of *Sternopygus dariensis* based on 12 PCGs.

Locality	Chucunaque	Tumaganti	Santa María
Tumaganti	0.02807		
Santa María	0.02845	0.00366	
San Pablo	0.02839	0.00354	0.00049

**Table 3:** Pairwise uncorrected p-distances between *Sternopygus* species based on *cox1* gene.

Species	*S. dariensis*	*S. arenatus*	*S. xingu*
*S. arenatus*	0.06599		
*S. xingu*	0.12034	0.10404	
*S. macrurus*	0.14441	0.12422	0.13509

TCS^35^ haplotype networks were characterized by two centrally shared haplotypes, corresponding to the eastern and western populations, which were separated by up to 13 mutational events. In addition, there were three peripheral haplotypes. The most common haplotype was shared by the western populations (Santa María and San Pablo) and represented up to 45% of all sampled individuals. The second-most common haplotype was unique to the eastern population (Chucunaque River; Supplementary Fig. S2). These results suggest the presence of two distinct genetic groups spanning the four geographical locations sampled.

### Time to the most recent common ancestor (TMRCA)

**Figure 4:**
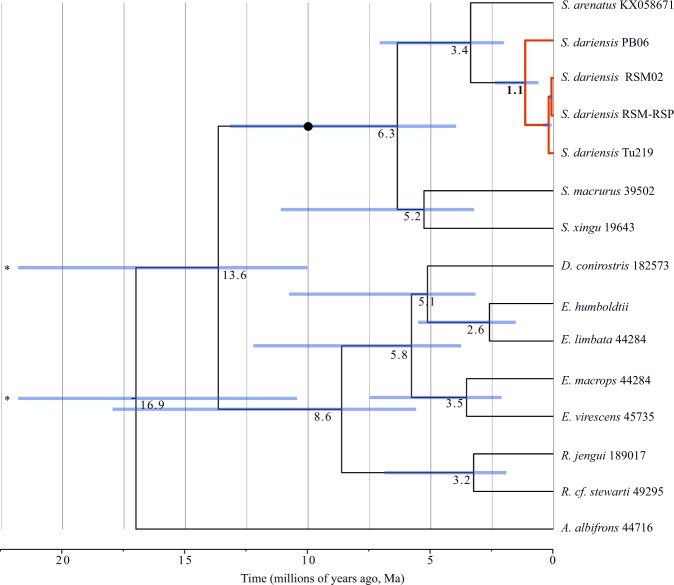
Divergence time estimates among *Sternopygus* species. Results are based on 2217 bp of concatenated *cox1*, *cytb* and *16S* with posterior probabilities from Bayesian Inference (BI) shown in grey, reconstructed using BEAST. Nodes are individually numbered, with gray bars indicating 95% confidence intervals on divergence time estimates (in My before present). Calibrated node is indicated with a black circle. Asterisks indicate that confidence interval bars are truncated.

Mitochondrial-based dating of TMRCA placed the first split within Sternopygidae around 13.6 Ma (95% HPD: 10–28.4 Ma; Fig. 4), separating members of Eigenmanninae and *Sternopygus*. Two clades separated 6.3 Ma (95% HPD: 2.58–12.28 Ma) were identified within *Sternopygus*, the first one including *S. arenatus* and *S. dariensis*; the second clade included *S. macrurus* and *S. xingu*. The split within the first clade (*S. arenatus* and *S. dariensis*) occurred during the Pliocene period, approximately 3.4 Ma (95% HPD: 1.2–6.7 Ma), while divergence between western and eastern clades of *S. dariensis* took place in the Pleistocene, approximately 1.1 Ma (95% HPD: 0.5–2.6 Ma).

## Discussion

Allopatric divergence most often occurs when populations accumulate random genetic differences as a byproduct of spatial isolation^19,36,37^. However, the extent to which spatially segregated populations show progress towards allopatric divergence is not always obvious. Here, we explore this issue by using a panel of over 285 UCE, SNPs and complete mitogenome sequences in the weakly electric fish *S. dariensis* in the context of the biogeographic history of the Isthmus of Panama.

Overall, our analysis of population structure based on UCEs revealed two divergent clusters (without evidence for admixture) that corresponded to eastern (i.e., Chucunaque/Tuira) and western populations (Santa María, San Pablo and Tumaganti; Fig. 1B). Indeed, over 77% of UCE loci, distributed across 22 of 29 chromosomes of the channel catfish and across available scaffolds of the electric eel, contained SNPs differentially fixed between these two genetic clades (Fig. 2). In addition, the frequency distribution of *F*_ST_ estimates across the entire dataset was highly skewed toward large values (Fig. 2). Thus, our results are consistent with a pattern of genetic differentiation under strict geographic isolation (i.e., a model of allopatric divergence^38^) and suggest that *S. dariensis* is composed of two genetic lineages, likely representing incipient allopatric species.

### Tempo and mode of allopatric divergence in *S. dariensis*

Allopatric divergence in primary freshwater fishes, including weakly electric fishes, in the context of Lower Mesoamerica is often associated with multiple colonization events during the dynamic formation of the Isthmus of Panama^6,12^. However, we showed that substantial progress towards allopatric speciation could also occur *in situ* following the closure of the Isthmus. This was supported by our phylogenetic analysis, which indicated that the Isthmian populations of *S. dariensis* constitute a monophyletic group (Fig. 3), likely derived from the South American *S. arenatus* over 3 Ma ago (Fig. 4). Thus, allopatric divergence in *S. dariensis* is likely the result of a single colonization event, followed by segregation of populations via colonization and dispersal. In addition, our analyses of mitogenome sequences revealed that divergence between eastern and western populations of *S. dariensis* is substantial (2.8%; Table 2) when compared to divergence across established *Sternopygus* species (6.6% between *S. dariensis* and *S. arenatus*; Table 3). This suggests an early geographic split in the mitochondrial genome between these populations, which occurred ~1.1 Ma (Fig. 4).

The historical context of this divergence is consistent with a scenario of early divergence between populations from Tuira/Chucunaque and Tumaganti watersheds, followed by more recent dispersal from Tumaganti to the western watersheds (Santa María and San Pablo). This scenario could explain the low genetic variation across the three western populations (i.e., Tumaganti, Santa María and San Pablo), possibly due to a strong bottleneck (or founder effect) or a limited time for the accumulation of genetic differences^39–41^. This expansion and contraction of populations was likely facilitated by changes in sea level during the Pleistocene glaciation oscillations^42^. For instance, during periods of low sea level, large portions of the eastern Pacific seabed remained exposed^43–45^, likely facilitating the exchange of freshwater fishes between eastern and western watersheds^39,44,46^. By contrast, during periods of high sea level, riverine populations likely became spatially isolated due to the intrusion of seawater^39,47,48^. It is important to notice that analyses of behavioral and genetic incompatibility may be necessary to confirm if these allopatric populations represent reproductively isolated populations/species within *S. dariensis*. For example, previous studies on electric fishes have highlighted the role of the temporal (shape) properties of the EODs in maintaining prezygotic isolation and speciation^49,50^. Cytogenetic analyses have also been used to test for genetic incompatibility Gymnotiformes^51,52^. Overall, however, our findings indicate that substantial genetic divergence has accumulated within the Isthmus of Panama. This also suggests that the diversity of *Sternopygus* species might have been underestimated, possibly due to the existence of cryptic species across the Isthmian range of the genus.

### Exploring the genetic consequences of allopatric divergence in *S. dariensis*

An expected consequence of allopatric divergence is the accumulation of genetic differences across large portions of the species’ genome due to random drift^38,53,54^. However, allopatric divergence may also be affected by adaptive processes (e.g., local adaptation^55,56^), which might lead to genomic islands of divergence^57,58^. Distinguishing between these possibilities requires a large number of genetic markers that can be mapped across the genome^59,60^. Although our analysis only included a few individuals per population and a reduced representation of the species’ genome (285 SNPs), the high number of fixed loci between populations, and the broad distribution of these loci across the majority of the species’ chromosomes, is consistent with a model of strict allopatric divergence^38,53^. In addition, the low number of loci under selection also points towards the role of non-adaptive processes as the predominant driver of divergence in *S. dariensis*. For instance, Picq *et al*.^6^ found sizeable evidence of the role of drift in EOD evolution. However, further work is needed to disentangle the contribution of both factors to *in situ* divergence in *S. dariensis* post-colonization of the Isthmus of Panama. This work could benefit from inclusion of additional sampling sites, and a larger number of individuals to examine genetic structure across the entire range of the species. In addition, future work should explore the geographic origins of *S. dariensis* in South America and its pattern of colonization of the Isthmus of Panama.

In summary, our results suggest that allopatric divergence in *S. dariensis* progressed *in situ* post-colonization of the Isthmus of Panama, and that genetic divergence is likely to occur widely across the species’ genome. Thus, our findings support the pattern of genetic differentiation expected under the classical model of allopatric divergence. Although reproductive isolation between allopatric populations is difficult to determine in nature, we suggest that integrating UCEs and mitogenome data is a useful approach to assess the evolution of genetic incompatibilities between geographically isolated populations. Our findings also underscore the role of vicariance and spatial isolation in promoting diversification in weakly electric fishes. Although more work is needed to disentangle the demographic and phylogeographic history of *S. dariensis* across its range, the study of spatially segregated populations within the context of the Isthmus of Panama could provide further insights into the accumulation of genetic differences that drive allopatric diversification in freshwater fishes.

## Methods

### Taxon sampling and ethical statement

Fish were first detected using a wire electrode connected to a mini-amplifier (Radioshack, Fort Worth, TX), and then collected using a dip-net. Fish were then euthanized with an overdose of eugenol (C_10_H_12_O_2_) derived from clove oil. Twelve specimens of *Sternopygus dariensis* were collected from four sites in the Republic of Panama (Fig. 1A): San Pablo River (SP: 8°11ʹ24ʺ N, −81°15ʹ0ʺ W), Santa María River (SM: 8°13ʹ50ʺ N, −80°58ʹ12ʺ W), Tumaganti River (TU: 9°13ʹ12ʺ N, −78°52ʹ47ʺ W) and Quebrada La Hoya stream, which flows into the Chucunaque River (PB: 8°15ʹ0ʺ N–77°43ʹ11ʺ W). Two individuals of *Eigenmannia humboldtii* were also sampled and used as outgroups in the phylogenetic analysis.

### UCE library preparation and sequencing

We extracted DNA from frozen-preserved tissues following the Qiagen DNeasy Blood and Tissue Kit (Qiagen, Valencia, CA.) protocol. We quantified all DNA extracts with a Qubit Fluorometer (Life Technologies, Inc.), assessed each extract for quality by electrophoresis, and then sheared extracts by sonication to a target size of 600 bp on a Covaris S220 instrument (Covaris, Woburn, Massachusetts, USA). Approximately, 500 ng of genomic DNA was used to prepare twelve 300 bp paired-end libraries, following the protocol Kapa Kit (New England Biolabs, Ipswich, MA, USA), and the UCE protocol described in Faircloth *et al*.^61^, available online from http://ultraconserved.org. We enriched libraries for UCE targets using Arbor Biosciences UCE Capture Kits (myBaits UCE Actinopterygians 0.5Kv1) designed to target 500 highly conserved loci across fishes. We determined the size of enriched and purified pools with a Bioanalyzer (Agilent Technologies, Inc.), and quantified enriched libraries by qPCR (Kapa Biosystems) prior to sequencing. Genomic libraries were multiplexed before sequencing 300 bp from both ends on the Illumina MiSeq platform at Naos Molecular Laboratory of the Smithsonian Tropical Research Institute (STRI), in Panama City, Panama.

### UCEs bioinformatics

Raw sequence data were converted to FASTQ before demultiplexing, trimming and cleaning using Illumiprocessor^62^, which works with Trimmomatic^63^. We then followed the standard PHYLUCE^64^ pipeline (http://phyluce.readthedocs.io/en/latest) for processing target-enriched UCEs data. Trinity version r2013-02-25^65,66^ was used to assemble reads using the script *assemblo_trinity.py*. We then used the *match_ contigs_to_probes.py* script to map assembled contigs to the UCE probes, which allowed us to assemble contigs representing enriched UCE loci from each species. We created two FASTA datasets: the first containing both the ingroup and outgroup taxa, and the second with only the ingroup, to increase the number of shared loci. We then followed the PHYLUCE pipeline to produce MAFFT^67^ alignments across all loci from both datasets. We removed locus names from each alignment, and created a complete dataset, with each locus containing data for all the individuals. Alignments of the first dataset (containing both ingroup and outgroup) were concatenated in PHYLIP format for subsequent analyses of phylogenetic structure (see below).

We chose the sample with the most UCE contigs recovered within the ingroup as reference sequence to call SNPs. We mapped reads (per individual) to this reference using the program BWA^68^. We converted SAM files to Binary Alignment Map (BAM) files format using the SAMtools^69^, and Picard (http://broadinstitute.github.io/picard/) to identify and remove PCR duplicates. We added read groups for each individual using Picard, and merged the BAM files across individuals with the SAMtools. We used the Genome Analysis Toolkit (GATK; McKenna *et al*.^70^ to identify and realign indels, to call and annotate SNPs and indels, and to mask SNP calls around indels, following the population genomics pipeline for UCEs developed by Faircloth and Harvey (https://github.com/mgharvey/seqcap_pop). This included restricting data to high-quality SNPs (Q30), and read-back phasing in GATK. At the end of the pipeline we created a Variant Call File (VCF) with nuclear SNPs. Finally, we used Python scripts from the seqcap_pop pipeline to convert the phased VCF into an input file for subsequent population analyses.

### UCE analyses

To quantify phylogenetic structure across species (i.e., the first dataset), we performed unpartitioned concatenated maximum-likelihood (ML) analyses, using RAxML 8.0.19^71^. Support for the best ML topology was assessed by performing 1000 nonparametric bootstraps in CIPRES Science Gateway^72^. For this analysis we used the GTR GAMMA site-rate substitution model for the best ML tree obtained from CIPRES Science Gateway^72^, using JModelTest 2^73^.

To estimate population structure across *S. dariensis* populations, we used a Bayesian clustering algorithm as implemented in STRUCTURE 2.3.4^74^. One random SNP from each locus was selected using the script *structure_from_vcf.py* (github.com/mgharvey/seqcap_pop) to create STRUCTURE input file, to minimize linkage disequilibrium. The number of subpopulations (ΔK) was determined using the ad-hoc statistical method, based on the rate of change in the log probability of data between successive K values. Ten independent runs for K values ranging from 1 to 4 were performed with a burn-in length of 50,000, followed by 500,000 interactions. Best K results were analyzed on Structure Harvester^75^ and Clumpak^76^.

To further examine divergence across *S. dariensis* populations, we quantified the proportion of SNPs that were differentially fixed (*F*_ST_)^77^ between the two major clades recovered from the STRUCTURE analysis (see results). These analyses were performed in the R package adegenet version 3.2.2^78,79^, and was performed on the 285 SNPs data set using the script *adegent_from_vcf.py* (github.com/mgharvey/seqcap_pop). To determine the genomic distribution of differentiated SNPs, we mapped each fixed SNP (UCE locus) to the available scaffolds of the electric eel, *Electrophorus electricus*^80^ genome using BLAST on SequenceServer (http://www.sequenceserver.com) implemented in EFISH genomics (https://efishgenomics.integrativebiology.msu.edu/blast_search/). We also mapped the fixed SNPs to the channel catfish, *Ictalurus punctatus*^81^ chromosomes, using the NCBI Genome Workbench version 2.12.8. In cases in which we obtained multiple hits, we retained the hits with >90% sequence identity and the highest query coverage. Finally, to examine the pattern of genetic divergence between clades, we estimated the frequency distribution, including skewness, of *F*_ST_ values across the entire SNP dataset.

### Outlier analyses

We quantified outlier loci with two methods: BayeScan 2^33^ and PCAdapt^82^. BayeScan uses differences in allele frequencies between populations, and estimates the probability that each locus is subject to selection using a Bayesian method. BayeScan was run under default settings. In PCAdapt, population structure is defined with PCA, and outliers are detected with respect to their contribution to population structure. Cattell’s graphical rule was used to choose the number of principal components (K) that identify potential SNPs under selection. Outliers were selected by performing the *q*-value procedure at a false discovery rate (FDR) of 0.05 using the R package qvalue^83^. PCAdapt was run assuming three genetic clusters after graphical evaluation of the eigenvalues according to Luu *et al*.^82^. To account for population structure, we retained the first two PC axes that explained most variation. For both analyses, the input file was created using the previously generated VCF file, and it was converted to other formats, as needed, using PGD Spider^84^ for BayeScan and Plink v1.9^85^ for PCAdapt.

### Recovery of mitogenomes

We identified mitogenomes from UCEs *off-target* reads by following the same methodology described in Aguilar *et al*.^34^. The complete sequences of mitogenomes were annotated in Geneious version 11.1.4^86^ using the complete mitochondrial genome sequence of *S. dariensis* (GenBank accession no. MH399590) as a reference.

### Phylogenetic analyses of complete mitochondrial genomes

In order to compare mitochondrial gene sequences, we extracted the protein coding genes (*nad6* and stop codons excluded). We aligned them using Multiple Alignment using Fast Fourier Transform (MAFFT)^87^ in Geneious version 11.1.4^86^. *Sternopygus arenatus*^88^ and *Eigenmannia sp*. mitogenomes^89^ were used as outgroup (Table 1). We manually checked the alignments to correct for annotation errors based on consensus, and removed positions with long gaps, as well as regions with uncertain alignment, ambiguous portions and stop codons. A General Time Reversible (GTR) model, with a proportion of invariable sites (I) and heterogeneity of substitution rates among sites using gamma distribution (G), was selected as the preferred model of nucleotide sequence evolution by jModelTest2 on XSEDE^73^, performed in the CIPRES Science Gateway^72^, with corrected Akaike information criterion (AIC). Maximum likelihood (ML) analyses were performed using RAxML. The resulting tree topology was evaluated by a rapid bootstrap analysis with 1000 replicates. Bayesian inference (BI) analyses were performed in MrBayes on XSEDE version 3.2.6 on CIPRES Science Gateway^72^. We ran two independent runs of 2,000,000 generations for each of the four chains. Each chain was sampled every 2000 generations with a burn-in of 25%. Trees inferred prior to stationarity were discarded as burn-in, and the remaining trees were constructed using a 50% majority-rule consensus tree with posterior probabilities.

We also assessed the evolutionary distance (uncorrected p-distance) among *S. dariensis* populations across all of the genes (concatenated) using uncorrected p-distances among unique haplotypes with MEGA version 7^90^. In addition, we calculated pairwise distance across *Sternopygus* species (*S. dariensis*, *S. arenatus*, *S. xingu* and *S. macrurus*), using the *cox1* gene. Standard error estimates were obtained by a 1000 bootstrap replicates under a maximum likelihood model^91^. Phylogenetic relationships between haplotypes were determined by constructing a Templeton, Crandall and Sing (TCS) haplotype network^35^ based on *cox1* sequences in PopArt 1.7 (Population Analysis with Reticulate Trees^92^). One previously published *cox1* (645 bp) sequence^6^ from Tuira River from Eastern Panama, was aligned independently to the newly generated mitogenomes.

### Time to the most recent common ancestor

We estimated divergence time among populations and species using BEAST2 on XSEDE v2.4.8^93^ on the CIPRES Science Gateway^72^ under a relaxed molecular clock with uncorrelated lognormal distribution of rates, to allow for lineage-specific rate variation, and using a Yule speciation model as tree prior. Sequences of *cox1*, *cytb* and *16S* were concatenated on a 2217 bp alignment. Nucleotides were first grouped into four different partitions: the 1st, 2nd, and 3rd codon positions respectively for *cox1* and *cytb*; and the *16S*. Nucleotide substitution models were estimated for each partition using the Bayesian Information Criterion in Partition Finder^94^. The best-fit models for the three genes were TVM + I + G (for the *16S* region, and the 1st and 2nd codon positions), and the TRN + G (for the 3rd codon position of the coding genes). Bayesian posterior distributions of evolutionary rates were estimated using Markov Chain Monte Carlo (MCMC) procedure. Four independent MCMC chains were run for 4 × 10^7^ generations and were sampled every 1,000 generations, after discarding the first 20% as burn-in. We used TRACER version 1.6^95^ to test for convergence of the chains to the stationary distribution, which was determined by an effective size (ESS) of over 200^95^. The four independent runs were combined using LogCombiner v1.8, and the dates of divergence along with their 95% confidence intervals (HPD) were estimated using Tracer v1.6. The resulting phylogeny and the 95% HPD for the dates of divergence for the major clades were visualized using FigTree v1.4^96^.

To calibrate the molecular clock, we used a fossil of *Humboldtichthys kirschbaumi* dated to c. 10 Ma, which shares morphological characters (i.e., opercle) with extant *Sternopygus* species^97^. The fossil age was placed on the stem node of the genus *Sternopygus*, using an uniform prior distribution with a maximum age equal to the maximum tree root height, following Picq *et al*.^6^. We used the three sequences (*cox1*, *cytb* and *16S*) to include all *Sternopygus* species available in Genbank and four genera of Gymnotiformes as outgroups (Supplementary Table S2).

### Ethics statement

Sampling permit was obtained from the Panamanian Ministry of Environment (Permit number SE/A-100-14). This research was approved by the Institutional Animal Care and Use Committee (IACUC) at the Instituto de Investigaciones Científicas y Servicios de Alta Tecnología (INDICASAT AIP), and all methods were performed according to the guidelines and regulations of the approved protocol (IACUC-16-001).

## Supplementary information

Supplementary information
