# Supplementary material for: Tempo and mode of allopatric divergence in the weakly electric fish Sternopygus dariensis in the Isthmus of Panama
Source: Sci Rep. 2019 Dec 11;9:18828. doi: 10.1038/s41598-019-55336-y (PMC6906317; doi:10.1038/s41598-019-55336-y)
Supplement: Supplementary file 1 — Supplementary information [file 41598_2019_55336_MOESM1_ESM.pdf]

Supplementary Information

Tempo and mode of allopatric divergence in the weakly electric fish *Sternopygus dariensis* in the Isthmus of Panama

Celestino Aguilar[1,2,3,4], Matthew J. Miller[1,5], Jose R. Loaiza[1,3,6], Rigoberto González [3], Rüdiger Krahe[7,8] and Luis F. De León[1,3,4,*]

[1] *Centro de Biodiversidad y Descubrimiento de Drogas, Instituto de Investigaciones Científicas y Servicios de Alta Tecnología (INDICASAT AIP), P. O. Box 0843-01103, Panama, Republic of Panama*

[2] *Department of Biotechnology, Acharya Nagarjuna University, Guntur, India*

[3] *Smithsonian Tropical Research Institute, P.O. Box 0843-03092, Balboa Ancón, Panama, Republic of Panama*

[4] *Department of Biology, University of Massachusetts Boston, Boston, MA, USA*

[5] *Sam Noble Oklahoma Museum of Natural History and Department of Biology, University of Oklahoma, Norman, OK, USA*

[6] *Programa Centroamericano de Maestría en Entomología, Universidad de Panamá, Republica de Panama*

[7] *Institut für Biologie, Humboldt-Universität zu Berlin, Berlin, Germany*

[8] *Department of Biology, McGill University, Montreal, QC, Canada*

* For correspondence: LFD – Department of Biology, University of Massachusetts Boston, Boston, MA, USA, email: luis.deleonreyna@umb.edu

Supplementary Table S1. Genomic distribution of highly differentiated UCE SNPs between western and eastern populations of *Sternopygus dariensis* in Panama. Fixed SNPs were mapped to both the electric eel (*Electrophorus electricus)* scaffolds, and the channel catfish (*Ictalurus punctatus*) chromosomes.

Locus	Position of fixed SNP	Electric eel			Channel catfish		
		Scaffold	Start	End	Chromosome	Start	End
uce-392	106	eel_0.2-scaffold360	62458	61665	1	2265835	2266448
uce-61	547	eel_0.2-scaffold405	30382	29691	1	32578797	32579167
uce-311	670	eel_0.2-scaffold498	244959	244127	1	23063743	23064351
uce-750	863	eel_0.2-scaffold9	346243	347265	2	3811771	3812025
uce-711	708	eel_0.2-scaffold12	2116348	2115271	3	20529519	20529299
uce-103	211; 575	eel_0.2-scaffold168	336556	337523	3	23597204	23596561
uce-444	19	eel_0.2-scaffold262	487343	488311	3	16954131	16954447
uce-1053	265	eel_0.2-scaffold30	1503200	1504112	3	29332885	29333151
uce-834	557; 680	eel_0.2-scaffold470	240963	241889	3	15125596	15126148
uce-399	172	eel_0.2-scaffold470	111619	112661	3	15294078	15294456
uce-335	62	eel_0.2-scaffold485	148519	147718	3	32956017	32955459
uce-358	786	eel_0.2-scaffold67	1218908	1218016	4	23218528	23217994
uce-682	190	eel_0.2-scaffold82	1159980	1161096	4	23466331	23467101
uce-568	335; 356	eel_0.2-scaffold82	1230212	1231223	4	23394667	23394944
uce-811	498	eel_0.2-scaffold82	1223801	1224298	4	23400127	23399786
uce-3	35; 177	eel_0.2-scaffold82	970609	971660	4	15056180	15055754
uce-509	921	eel_0.2-scaffold10	1514538	1513445	6	30460624	30461727
uce-63	624	eel_0.2-scaffold11	2010519	2009662	6	13089832	13090439
uce-201	56; 80	eel_0.2-scaffold11	2007054	2006204	6	13093052	13093857
uce-987	17; 393	eel_0.2-scaffold289	295634	296059	6	9309419	9309798
uce-526	514	eel_0.2-scaffold289	31135	31618	6	9631385	9631555
uce-185	498; 949	eel_0.2-scaffold289	152997	153982	6	9478067	9478897
uce-1064	212	eel_0.2-scaffold289	88852	89795	6	9562169	9561639
uce-997	714	eel_0.2-scaffold83	338038	338785	6	30659055	30659595
uce-653	851	eel_0.2-scaffold83	295115	295983	6	30605916	30606595
uce-143	143; 161	eel_0.2-scaffold83	345739	346816	6	30666926	30667484
uce-642	332; 415; 499; 544	eel_0.2-scaffold83	309730	310514	6	30624973	30625424
uce-701	230; 881; 920	eel_0.2-scaffold192	5499965	5500169	7	25447905	25448088
uce-435	108; 148; 488	eel_0.2-scaffold683	66165	66834	7	27746063	27746373
uce-1080	574; 1112	eel_0.2-scaffold13	772812	773869	8	8085463	8085039
uce-1027	742	eel_0.2-scaffold13	670084	669265	8	8232744	8233414
uce-535	339	eel_0.2-scaffold146	16731	15654	8	6860847	6860998
uce-160	59	eel_0.2-scaffold212	372068	371137	8	10632136	10633074

uce-106	659; 804	eel_0.2-scaffold40	596617	596030	8	24229151	24229281
uce-982	290; 686; 816	eel_0.2-scaffold1287	27974	27031	9	29316297	29316490
uce-410	2	eel_0.2-scaffold174	397196	396231	9	19955047	19954417
uce-375	307	eel_0.2-scaffold174	157494	156715	9	19686104	19685644
uce-183	67; 192; 649; 746	eel_0.2-scaffold174	112214	111447	9	19635641	19635138
uce-776	709	eel_0.2-scaffold489	238792	239801	9	20442448	20443077
uce-416	543	eel_0.2-scaffold578	141119	140428	9	377089	377216
uce-693	138	eel_0.2-scaffold649	12812	13740	9	5148903	5148288
uce-372	507	eel_0.2-scaffold92	706289	705552	9	15300347	15300817
uce-227	319	eel_0.2-scaffold92	971219	970327	9	14999507	14999868
uce-1316	99	eel_0.2-scaffold92	730636	729684	9	15268926	15269876
uce-332	25	eel_0.2-scaffold154	740712	741750	10	12547603	12548292
uce-356	415; 421	eel_0.2-scaffold721	164305	165088	10	28469038	28469355
uce-636	806	eel_0.2-scaffold89	28609	29867	10	21157996	21158199
uce-717	198	eel_0.2-scaffold119	46819	45947	11	12566769	12567573
uce-1266	674	eel_0.2-scaffold2	1959327	1959906	11	20110431	20110653
uce-1321	6	eel_0.2-scaffold392	34785	33697	11	11833856	11833505
uce-666	898, 968	eel_0.2-scaffold52	813537	812438	12	18328802	18329121
uce-863	847	eel_0.2-scaffold173	409460	410389	13	7336264	7336592
uce-805	677; 678	eel_0.2-scaffold173	248278	249136	13	7157630	7158500
uce-944	156; 450	eel_0.2-scaffold47	188353	187480	14	9845885	9846133
uce-1293	50	eel_0.2-scaffold47	188353	187480	14	12010636	12010158
uce-89	392	eel_0.2-scaffold184	620423	620656	15	4222601	4222799
uce-605	293; 354	eel_0.2-scaffold270	181844	182951	15	19136275	19137097
uce-191	373; 735	eel_0.2-scaffold270	103469	104394	15	19225541	19226068
uce-175	358; 832	eel_0.2-scaffold270	222457	223551	15	19080643	19081162
uce-1238	127	eel_0.2-scaffold270	61572	62471	15	19304766	19304591
uce-167	931	eel_0.2-scaffold291	283753	282725	15	18036469	18037077
uce-276	348; 537	eel_0.2-scaffold482	166591	167220	15	16665411	16665884
uce-1242	52	eel_0.2-scaffold1	975919	975467	18	5688527	5688926
uce-1168	153; 154; 423; 580; 728	eel_0.2-scaffold62	342081	342833	18	4458918	4458762
uce-864	171; 669; 741; 788	eel_0.2-scaffold347	186491	185428	19	13703053	13702781
uce-1173	671	eel_0.2-scaffold347	290491	289713	19	13848469	13849059
uce-496	229	eel_0.2-scaffold367	387137	386351	19	18782137	18781809
uce-305	295	eel_0.2-scaffold946	11421	12093	19	19962703	19962372
uce-1307	835	eel_0.2-scaffold241	365196	366052	20	2795071	2795313
uce-124	733; 810	eel_0.2-scaffold3	1867393	1866333	20	21610715	21610895
uce-968	162	eel_0.2-scaffold539	103889	104596	22	12430080	12431000
uce-1175	258	eel_0.2-scaffold539	165593	166565	22	12515661	12516194

uce-518	416; 812	eel_0.2-scaffold565	24368	25420	22	337740	338024
uce-975	103	eel_0.2-scaffold6	1361227	1361800	22	6732070	6732459
uce-651	622	eel_0.2-scaffold86	975886	974931	22	13091946	13092485
uce-934	681	eel_0.2-scaffold1150	64101	63204	23	3967450	3966902
uce-359	323; 324; 524	eel_0.2-scaffold136	520710	521384	23	14576000	14576187
uce-1192	856	eel_0.2-scaffold136	545441	546163	23	14534874	14535124
uce-529	333	eel_0.2-scaffold80	582462	581736	23	7901051	7901275
uce-1228	238	eel_0.2-scaffold420	177322	178283	24	13695997	13696442
uce-733	952	eel_0.2-scaffold69	1043316	1044404	25	16407702	16407371
uce-166	110; 239; 666	eel_0.2-scaffold312	335311	334248	27	4153272	4153704
uce-590	213; 1021	eel_0.2-scaffold314	196040	194892	27	5230670	5230873
uce-836	73; 160; 186; 612; 711	eel_0.2-scaffold141	156715	157413	--	--	--
uce-762	164	eel_0.2-scaffold192	424484	425040	--	--	--
uce-159	125; 393; 437; 1076	eel_0.2-scaffold225	584824	583715	--	--	--
uce-1188	27; 28; 215; 836	eel_0.2-scaffold237	203511	202561	--	--	--
uce-113	188	eel_0.2-scaffold383	313188	312506	--	--	--
uce-504	861	eel_0.2-scaffold432	242184	243266	--	--	--
uce-572	174	eel_0.2-scaffold547	254917	255172	--	--	--
uce-247	143; 346; 692; 921	eel_0.2-scaffold607	217353	218295	--	--	--

Supplementary Table S2. List of mtDNA *16S*, *cytb* and *cox1* genes sequences of Gymnotiformes retrieved from GenBank and database accession number.

Species	GenBank		
	16S	***cytb***	***cox1***
Sternopygus macrurus 39502	KR260159	KR491722	KR491580
Sternopygus xingu 19643	KR260161	KR491721	KR491582
Distocyclus conirostris 182573	KR260147	KR491726	KR491586
Eigenmannia macrops 44284	KR260150	KR491729	KR491589
Eigenmannia virescens 45735	KR260154	KR491730	KR491594
Rhabdolichops jegui 189017	KR260158	KR491737	KR491583
Rhabdolichops cf. stewarti 49295	KR260157	KR491725	KR491584
Apteronotus albifrons 44716	KR260172	KR491648	KR491538
	**Complete mtDNA**		
Sternopygus arenatus	KX058571		
Eigenmannia humboldtii	MH263668		
Eigenmannia limbata	MH263669		
Apteronotus rostratus	MH399592		

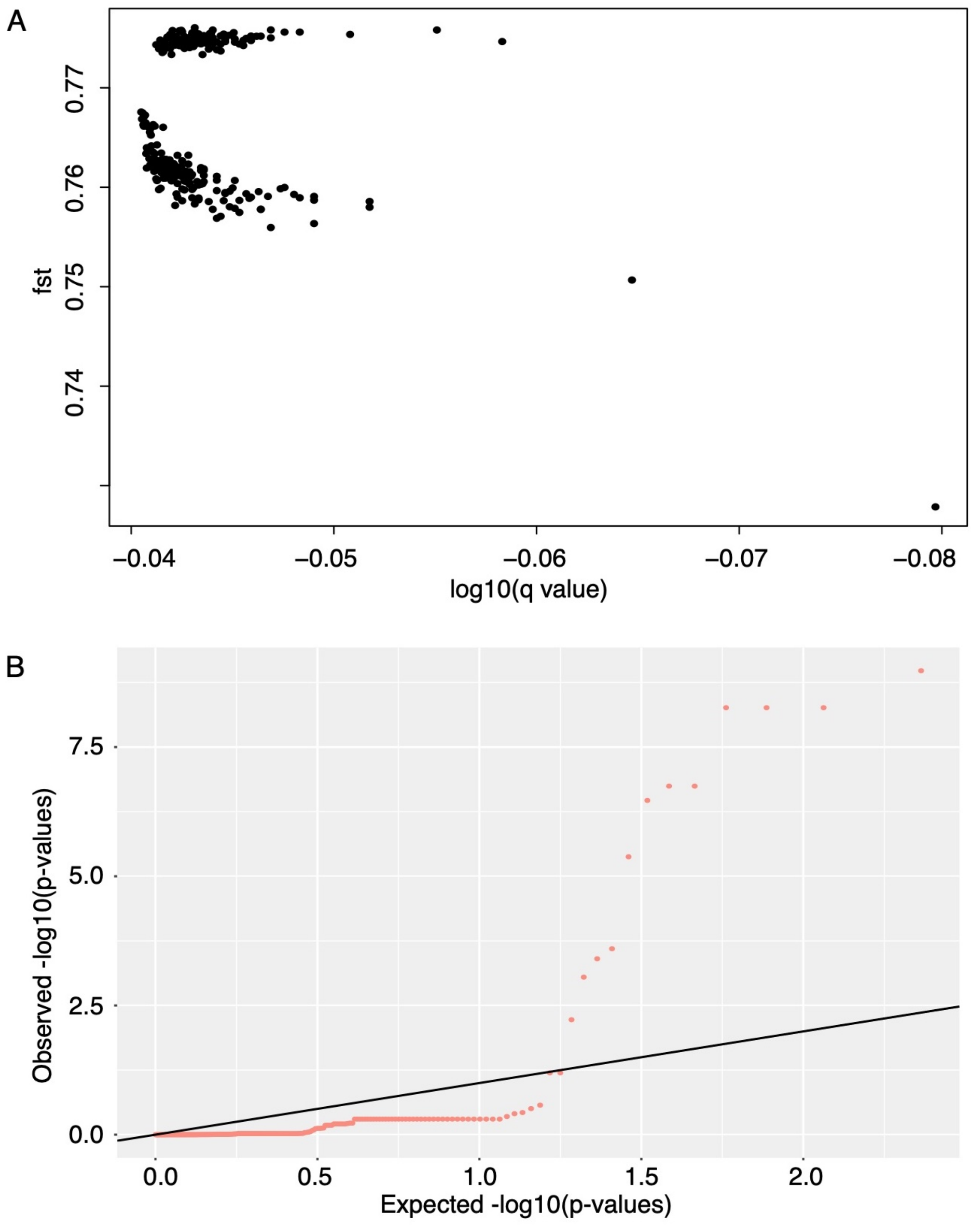

Supplementary Figure S1. Analysis of outlier loci between populations of *Sternopygus dariensis* using Bayesian-based BayeScan (A) and PCAdapt (B). The BayeScan plot (A) plot shows Fst against log10 (q-value), which is the FDR analog of the p-value. The Q-Q plot (B) from the PCAdapt analysis shows the observed and expected p-values (log10 transformed) with the black line showing the expected relationship at 0.05 significance threshold.

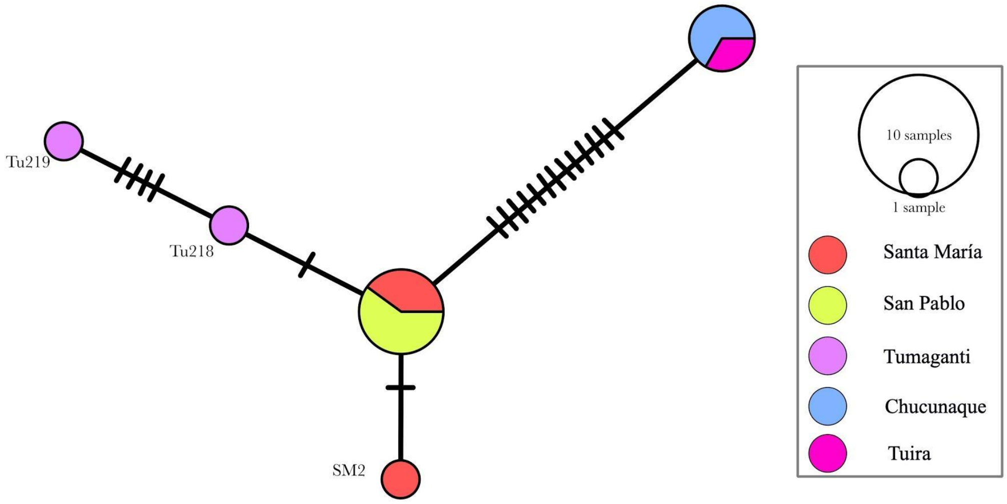

Supplementary Figure S2. Mitochondrial (*cox1*) haplotype network constructed from samples of *Sternopygus dariensis* using the TCS method as implemented in PopArt. The areas of the circles are proportional to the number of samples sharing each haplotype. Small ticks on branches indicate the number of mutations separating haplotypes. Each colour represents haplotypes found in a river.
